# The impact of petrochemical industrialisation on life expectancy and per capita income in Taiwan: an 11-year longitudinal study

**DOI:** 10.1186/1471-2458-14-247

**Published:** 2014-03-12

**Authors:** Ya-Mei Chen, Wan-Yu Lin, Chang-Chuan Chan

**Affiliations:** 1 Institute of Health Policy and Management, College of Public Health, National Taiwan University, Room 633, No. 17, Xuzhou Road, Taipei City 100, Taiwan; 2 Institute of Epidemiology and Preventive Medicine, College of Public Health, National Taiwan University, Room 501, No. 17, Xuzhou Road, Taipei City 100, Taiwan; 3 Institute of Occupational Medicine and Industrial Hygiene, College of Public Health, National Taiwan University, Room 722, No. 17, Xuzhou Road, Taipei City 100, Taiwan

## Abstract

**Background:**

Petrochemical industries have been identified as important sources of emissions of chemical substances, and adverse health outcomes have been reported for residents who live nearby. The purpose of the current study was to examine the adverse effects of petrochemical industrialization in Taiwan on the life expectancy and personal income of people living in nearby communities.

**Methods:**

This study compared life expectancies and personal income between one industrial county (Yunlin County) and one reference county (Yilan County), which had no significant industrial activity that might emit pollutants, in Taiwan through analysis of 11 year long and publicly available data. Data from before and after the petrochemical company in the industrial county started (year 1999) operating were compared.

**Results:**

Residents of the industrialized county had lesser increases in life expectancy over time than did residents of a similar but less-industrialized county, with difference means ranging from 0.89 years (p < 0.05) to 1.62 years (p < 0.001) at different stages. Male residents were more vulnerable to the effects of industrialization. There were no significant differences in individual income between the two counties.

**Conclusions:**

Countries, including Taiwan and the U.S., embracing petrochemical industries now face the challenge of environmental injustice. Our findings suggested that life expectancy lengthening was slowed and income growth was stalled for residents living in the industrial communities.

## Background

In 2012 August, U.S. state regulators and the Obama administration tried to rescue Sunoco’s Philadelphia refinery by agreeing to loosen certain environmental restrictions. This action raised an environmental justice question of weighing 850 jobs against the health of people living nearby the refinery. Such environmental justice issues are faced by many developing countries—including Taiwan and the other three countries that were in the 1960s to 1990s termed the Four Little Dragons or Four Asian Tigers, and now also those countries grouped together as BRICS and ASEAN nations. These nations adopted industrialisation, especially of their petrochemical industries, to achieve the goal of economic growth [1]. In Taiwan, development of the petrochemical industry began in the past half century, and the country has experienced fast economic growth during that same period of time.

Petrochemical industries have been identified as important sources of emissions of a wide range of chemical substances, some of which have been recognized as environmental carcinogens [2]. Research studies have reported the health hazards of exposure to these chemicals and the adverse health effects on residents of environmental pollution resulting from the petrochemical industry [3-9]. Only very few studies reported no additional risk to human health [10]. Most of these studies were occupational exposure studies which focused on workers at the petrochemical facilities. However, it is not only workers, but also non-occupationally related populations living nearby, that have the potential to be exposed to a large set of chemicals, including some that are health hazards. People who live nearby petrochemical facilities may be exposed to pollution resulting from the facilities, but it remains unknown whether their life expectancy is affected by the emissions or other factors associated with industrialisation.

Economic growth and health are in most cases positively related [11-13]. However, Szreter [1] examined the relationships between industrialisation and health and concluded that all economic exchange entails health risks, and that industrialisation typically results in a particularly concentrated cocktail of such health risks. Previous studies have shown that industrial facilities are more likely to be located in more disadvantaged communities [14,15]. This further raises an environmental injustice issue; do people who live near these facilities benefit from the economic growth that arrives with industrialisation.

Researchers in Taiwan have considered the petrochemical industry to be the main source of industrial air pollution in Taiwan. Subsequent to several high-profile air-pollution events in Taiwan in 2010 and 2011, public concern regarding potential health hazards for residents living near petrochemical facilities has been elevated [7].

### Study purpose

The purpose of the current study was to examine the adverse effects of petrochemical industrialisation in Taiwan on health. The study also examined whether petrochemical industrialisation contributed to economic growth among people living in nearby communities. Studies investigating the effects of industrialisation on health have been limited due to lack of appropriate data, especially longitudinal data. The current study seized an opportunity to observe how petrochemical facilities influence local residents’ life expectancy and personal income by analyzing 11 years of data collected both before and after a petrochemical facility began operating in central Taiwan.

Using Taiwan’s national data, the current study examined (1) the long-term health influence of petrochemical facilities on local residents, by comparing the life expectancy of residents living within a county in which a petrochemical facility resides to the life expectancy of residents of a reference area that did not have significant local pollution sources within its borders; and (2) the economic influence of petrochemical facilities on local residents, by comparing the per capita income of residents living within a county in which a petrochemical facility resides to the per capita incomes of residents of a reference county that did not include any petrochemical facilities within its borders.

## Methods

### Study design and the two counties chosen for study

We used a comparative study design to estimate the impact of petrochemical industry on life expectancies and per capita income by observing two counties in Taiwan, one with a petrochemical facility and one with no significant industrial activity that might emit pollutants. We compared outcome variables within each county and between the two counties at time points both before and after the petrochemical facility began operating.

**Figure 1:**
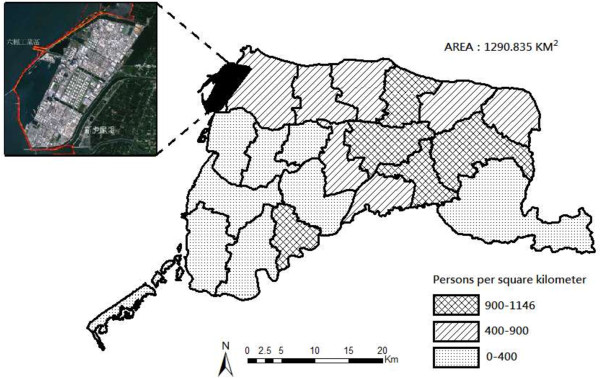
The petrochemical corporation in the industrialized county (Yunlin County).

We first selected Yunlin County as the industrialized county (referred to in this study as the industrialized county). Yunlin County has traditionally been an agriculturally based county in the western part of southern Taiwan, and has a population around 735,000 persons. A petrochemical industrial complex is located within this county (see Figure 1). The complex includes 66 facilities, including a naphtha cracking plant, petrochemical manufacturing, and coal-fired power plants. Since the Complex started operating in 1999, one study has pointed out that the concentrations of air pollutants such as PM10, ozone, and sulfur dioxide in Yunlin County have increased significantly [16]. The 2010 report of annual air pollutants emitted by the Complex indicated the following: particle pollutants, 3,340 metric tons; sulfur dioxide, 16,000 metric tons; Nox, 19,622 metric tons; and VOCs, 4,302 metric tons [16,17]. It is estimated that the Complex contributed to about 9.2% of Taiwan’s GDP in 2011 [18], which was increased significantly over the past 12 years since the Complex started operation [18,19]. Before settling down in the off-shore industrial zones in Yunlin County, the complex went through several options for selecting project sites due to local opposition in places such as Yilan County.

Yilan County (referred to in this study as the reference county) was very similar to the industrialized county in many ways, including its agricultural environment, and geographic proximity to the seashore. Yilan County had been first selected by the petrochemical complex company but then rejected by the Yilan County government in early 1990s. From 1996 to 2007, however, almost twice as many large manufacturing companies operated in the industrialized county as in the reference county. As a result, the current study uses Yilan County as a reference. See Table 1 for detailed information about these two counties.

**Table 1:** Descriptive information for Yunlin and Yilan counties from 1996 to 2007

**County**		**Yunlin (industrialized county)**				**Yilan (reference county)**		
Area (km^2^)^a^	1290.835				2143.625			
Year	1996–1998	1999–2002	2002–2004	2005–2007	1996–1998	1999–2002	2002–2004	2005–2007
Population (person)^a^	752,427	746,241	742,797	725,672	465,120	465,004	464,107	460,398
Number of large manufacturing^b^	35	42	48	42	22	21	25	22
Agricultural land (Hectare)^c^	112,870	112,364	111,992	112,550	58,265	158,641	164,438	200,826
Number of doctors (/1000 people)^a^	0.66	0.71	0.76	0.92	0.86	1.00	1.12	1.19
Number of hospital (/1000 people)^a^	0.031	0.032	0.029	0.026	0.030	0.032	0.026	0.026
Life expectancy at birth	74.05	74.88	75.51	76.16	74.84	75.8	76.88	77.65
Life expectancy at 5 years old	69.74	70.52	71.12	71.69	70.62	71.51	72.59	73.31
Life expectancy at 65 years old	15.98	16.63	17.17	17.78	16.59	17.05	17.94	18.64
Mortality rate (/10000)	671.41	611.04	681.14	751.94	698.77	684.57	695.4	744.42

Note. Large manufacturing indicating a company with more than 200 people.

Data source a. Ministry of the Interior. (URL: http://www.moi.gov.tw/stat/).

Data Source b. Small and Medium Enterprise Administration, Ministry of Economic Affairs (URL: http://www.moeasmea.gov.tw/mp.asp?mp=1).

Data Source c. Council of Agriculture, Executive Yuan (URL: http://www.coa.gov.tw/show_index.php).

Since the complex started operating in the industrialized county in 1999, studies have pointed out that the concentrations of air pollutants increased [16,17]. As for the reference county, instead of hosting a petrochemical facility, it has in the past decade continued to develop its agriculture industry as well as its sightseeing industry, with great effort toward maintaining the area’s natural beauty.

This study compared life expectancies and individual per capita income between the industrialized county and the reference county in Taiwan. Data from before and after the petrochemical facility in the industrial county started operating in 1999 were compared. Data from 1996 to 1998 were used as baseline data. Data collected from 1999 to 2007 were used to assess the effects of industrialisation, and findings were compared to the baseline data. To consider the phase-in effects of industrialisation, the study period was divided into four stages: (1) pre-industrialisation (1996–1998), (2) short-term industrialisation (1999–2001), (3) intermittent industrialisation (2002–2004), and (4) long-term industrialisation (2005–2007). As suggested in an environmental evaluation published by the Yunlin County government, the four stages of the study period were based on the complex’s manufacturing process and VOC (volatile organic compound) emission quantities [20].

### Data sources

Due to the availability and completeness of the data sources, we only investigated life expectancy and individual per capita income of the industrialized and reference counties. Life expectancy was evaluated at birth (referred as LE_birth_), at 5 years old (referred as LE_5_), and at 65 years old. Life expectancy (referred as LE_65_) data was collected from Life Tables for Taiwan on the website of the Department of Statistics in the Ministry of the Interior [21]. Individual income data was available from the website of the Execute Yuan, Directorate-General of Budget, Accounting, and Statistics [22]. Data from 1996 to 2007 were included for analysis.

### Analysis

The life expectancies in the reference county (Yilan County) and the industrialized county (Yunlin County) were examined longitudinally. Variables of interest (differences between the two counties in life expectancy for the total population, male residents, and female residents) and time were also analyzed using first-order autogression correlation structure. Life expectancy at birth (LE_birth_), at 5 years old (LE_5_), and at 65 years old (LE_65_) were all included for analysis. To further examine the impact of length of exposure to industrialisation, the life expectancies were compared cross-sectionally between the industrialized and the reference counties and examined at all four stages. Due to the concern of currency change over time, income per capita was compared only cross-sectionally. T-tests were employed to investigate the differences in life expectancy and per capita income between the two counties at four different stages. SAS version 9.1 was used for all analyses.

## Results

Both the industrialized county and the reference county experienced steadily increased life expectancy from 1996 to 2007. The LE_birth_ increased from 73.94 to 76.54 years and 74.52 to 77.94 years for the industrialized county and the reference county respectively. Since the patterns of increasing life expectancy were similar for life expectancies at different ages, the current study reports only life expectancy at 5 years old.

### Life expectancy discrepancies between counties

Using autoregression, the increase in life expectancy over time of the industrialized county was 0.084 (*p* < 0.0001), 0.088 (*p* < 0.0001), and 0.0368 (*p* < 0.01) years lesser than that of the reference county per year for LE_birth_, LE_5_, and LE_65._ respectively over the past 11 years (Table 2).

**Table 2:** Results of autoregression on life expectancy at birth, at 5 years old, and at 65 years old between the industrialized and reference counties

**Life expectancy at birth**	**All**		**Male**		**Female**	
	**Estimates**	***p*-value**	**Estimates**	***p*-value**	**Estimates**	***p*-value**
Intercept	73.8682***	< .0001	70.7387***	< .0001	77.5950***	< .0001
County	0.6765***	< .0001	0.8388***	0.0003	0.6056***	0.0003
Year	0.2329***	< .0001	0.1877***	< .0001	0.3045***	< .0001
County*Year	0.0843***	< .0001	0.1229***	0.0004	0.0050	0.8130
Life expectancy at age 5						
Intercept	69.5763***	< .0001	66.4387***	< .0001	73.3118***	< .0001
County	0.7546***	< .0001	0.9750***	< .0001	0.6094***	0.0007
Year	0.2164***	< .0001	0.1677***	< .0001	0.2926***	< .0001
County*Year	0.0883***	< .0001	0.1273***	< .0001	0.0095	0.6880
Life expectancy at age 65						
Intercept	15.7964***	< .0001	14.2856***	< .0001	17.2277***	< .0001
County	0.4633***	< .0001	0.7333***	< .0001	0.5823***	< .0001
Year	0.1981***	< .0001	0.1569***	< .0001	0.2436***	< .0001
County*Year	0.0368***	0.0070	0.0573***	< .0001	0.0009	0.9576

Note. The results are of the life expectancy of the reference county compared to the industrialized county. *p < 0.05; **p < 0.01; ***p < 0.001.

When the four stages of industrialisation were further analyzed and compared cross-sectionally, the LE_birth_ and LE_65_ in the reference county were higher and increased faster than the LE_birth_ and LE_65_ in the industrialized county in the four study stages, with difference means of 0.79 years (*p* < 0.05) at the pre-industrialisation stage, 0.92 years (*p =* 0.07) at the short-term industrialisation stage, 1.37 years (*p* < 0.01) at the intermittent industrialisation stage, and 1.49 years (*p* < 0.05) at the long-term industrialisation stage for LE_birth_; and difference means of 0.61 years (*p* < 0.05) at the pre-industrialisation stage, 0.42 years (*p =* 0.10) at the short-term industrialisation stage, 0.77 years (*p* < 0.05) at the intermittent industrialisation stage, and 0.86 years (*p* < 0.01) at the long-term industrialisation stage for LE_65_. The discrepancy of the mean differences between these two counties continued to increase over time. The patterns were even more prominent for LE_5_. The LE_5_ in the reference county was significantly higher and increased faster than the LE_5_ in the industrialized county in the four study stages, with the difference means of 0.89 years (*p* < 0.05) at the pre-industrialisation stage, 0.99 years (*p* < 0.05) at the short-term industrialisation stage, 1.47 years (*p* < 0.001) at the intermittent industrialisation stage, and 1.62 years (*p* < 0.001) at the long-term industrialisation stage (Table 3). This evidence could indicate that the impact of industrialisation on life expectancy could start 3–5 years after the facilities start running (intermittent industrialisation stage), and middle age adults may suffer more from the impact as time goes by.

**Table 3:** Differences of Life expectancies between the industrialized county and the reference county at all four stages

**Period**	**All**	**Male**	**Female**						
	**Difference Mean**	**( *SD *)**	***p*-value**	**Difference Mean**	**( *SD *)**	***p*-value**	**Difference Mean**	**( *SD *)**	***p*-value**
**Life expectancy at birth**									
Pre-industrialisation	0.79*	(0.24)	0.02	0.83*	(0.25)	0.02	0.85*	(0.25)	0.01
Short-term industrialisation	0.92	(0.45)	0.07	1.43*	(0.49)	0.02	0.27	(0.39)	0.45
Intermittent industrialisation	1.37**	(0.18)	<0.01	1.88***	(0.10)	<.0001	0.66	(0.35)	0.08
Long-term industrialisation	1.49*	(0.33)	0.01	1.93*	(0.35)	<0.01	0.76*	(0.30)	0.03
**Life expectancy at age 5**									
Pre-industrialisation	0.89*	(0.89)	0.02	1.01*	(0.27)	0.01	0.84*	(0.27)	0.02
Short-term industrialisation	0.99*	(0.39)	0.03	1.51*	(0.41)	0.01	0.33	(0.36)	0.33
Intermittent industrialisation	1.47**	(0.24)	<0.01	2.04***	(0.14)	<.0001	0.68	(0.41)	0.11
Long-term industrialisation	1.62**	(0.29)	<0.01	2.13**	(0.29)	<0.01	0.81**	(0.28)	0.02
**Life expectancy at age 65**									
Pre-industrialisation	0.61*	(0.22)	0.03	0.85**	(0.18)	<0.01	0.75*	(0.27)	0.03
Short-term industrialisation	0.42	(0.24)	0.10	0.84*	(0.25)	0.01	0.30	(0.25)	0.21
Intermittent industrialisation	0.77*	(0.26)	0.02	1.16**	(0.19)	<0.01	0.68*	(0.33)	0.07
Long-term industrialisation	0.86**	(0.18)	<0.01	1.33**	(0.14)	<0.01	0.62*	(0.21)	0.02

Note. Pre-industrialisation (1996–1998); Short-term industrialisation (1999–2001); Intermittent industrialisation (2002–2004); Long-term industrialisation (2005–2007).

*p < 0.05; **p < 0.01; ***p < 0.001.

**Figure 2:**
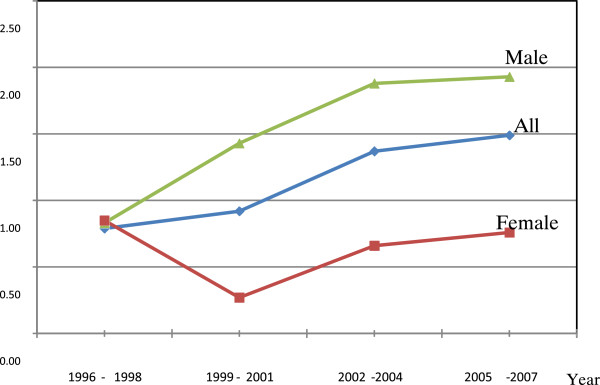
**The differences of life expectancy**_**5 **_**between the reference county (Yilan County) and the industrialized county (Yulin County) from 1996–2007 in Male and Female residents.**

We further conducted trend tests for the discrepancies in life expectancies between the industrialized county and the reference county, and our findings showed that the trends of life expectancy discrepancies between these two counties for all residents increased over time (*p* < 0.05) (Figure 2). This indicated the gap of life expectancy between the two counties increased over time.

### Life expectancy and gender differences

Using autoregression, the increase in life expectancy over time for male residents and female residents was different between the industrialized county and the reference county. Male residents in the industrialized county showed a smaller increase in life expectancy than the male residents living in the reference county, but there were no significant differences for female residents living in the two counties. The increase in life expectancy of the male resident in the industrialized county was 0.123 (*p* < 0.001), 0.127 (*p* < 0.0001), and 0.057 (*p* < 0.0001) years less than that of the reference county per year for LE_birth_, LE_5_, and LE_65_ respectively over the past 11 years (Table 2).

The gender effect was also noted, when life expectancy was compared cross-sectionally. The discrepancy of life expectancies between the reference county and the industrialized county was greater for the male population than for the female and whole population. The life expectancies of male residents in the reference county were significantly higher than the life expectancy of male residents in the industrialized county in all four study stages, with difference means of 0.83 years (*p* < 0.05) at the pre-industrialisation stage, 1.43 years (*p* < 0.05) at the short-term industrialisation stage, 1.88 years (*p* < 0.0001) at the intermittent industrialisation stage, and 1.93 years (*p* < 0.01) at the long-term industrialisation stage for LE_birth._ Again, the gap of life expectancy for male residents between the two counties increased over time. This pattern was similar for LE_5_ and LE_65_ (Table 3).

The female residents in the reference county had significantly higher life expectancies than female residents in the industrialized county in only two study stages, with difference means of 0.85 years (*p* < 0.05) at the pre-industrialisation stage, and 0.76 years (*p* < 0.05) at the long-term industrialisation stage for LE_birth_. The patterns were similar for LE_5_ and LE_65_ (Table 3). This evidence could indicate that the impact of industrialisation on life expectancy could start soon after the facilities start running (short-term stage) for male residents living the industrialized county, but it took longer (6–8 years) to manifest the impact for female residents.

We further conducted trend tests for the discrepancies in LE_5_ between the reference county and the industrialized county for male and female residents in these two counties respectively. Our findings showed that the trends of life expectancy discrepancies between these two counties for male residents significantly increased over time (*p* < 0.01), but did not increase for female residents (Figure 2). This indicated the gap of life expectancy for male residents between the two counties increased over time.

### Per capita income between counties

The per capita income of residents living in the industrialized county was not found to be significantly different from the individual’s per capita income of residents living in the reference county at any time period (Table 4). Residents living in the industrialized county did not economically benefit from having a chemical-intensive industry operating in their neighborhood.

**Table 4:** Comparisons of per capita income between the reference county (Yilan County) and the industrialized county (Yunlin County) from 1987 to 2007)

**Period**	**Per capita income**				
	**Yilan**	**Yulan**	**Difference mean**	**SD**	**p-value**
Pre-industrialisation	189,408	179,850	9,558	13,614	0.44
Short-term industrialisation	219,520	219,743	−222	11,229	0.98
Intermittent industrialisation	205,701	200,526	5,175	4,379	0.22
Long-term industrialisation	231,497	219,722	11,775	8,579	0.17

Note. Pre-industrialisation (1996–1998); Short-term industrialisation (1999–2001); Intermittent industrialisation (2002–2004); Long-term industrialisation (2005–2007).

## Discussion

Our findings indicate that local residents seemed not to benefit economically from hosting a petrochemical company in their county. They did, however, suffer negative effects on life expectancy increases over time. The impact of the industrialisation process on local residents’ life expectancy also indicated strong gender differences.

### Industrialisation and life expectancy

Although the industrialized county and the reference county have increased life expectancies from 1996 to 2007, the life expectancy in the reference county increased faster (by 0.084 years more each year) than did the life expectancy in the industrialized county, and the discrepancies increased over time. As previous literature has focused only on the adverse effect of the petrochemical industry on particular health problems, this study provides valuable information on the impact of a petrochemical industry on local residents’ life expectancies [3,9,23]. Our results also indicate that the impact of industrialisation on life expectancy could start 3–5 years after the facilities start running (intermittent industrialisation stage) for the whole population and the older population. For the adult population (life expectancy at 5 years old), who might be more likely to work in outdoor environment, may be more susceptible to the impact and suffer faster and more from the impact as the time goes by.

However, the mechanisms by which petrochemical industrialisation affects residents’ health were not fully understood, it is possible that the residents of the industrialized county suffered from air pollutants, possibly due to the local petrochemical facility, which resulted in more chronic illness and therefore smaller increases in life expectancy rates than in the reference county. Most of the previous literature reported negative effects of petrochemical emissions on neighboring residents’ health, which might explain the smaller increase in life expectancies in the industrialized county [3-9,24,25].

### Industrialisation and gender effects

The current study’s findings showed that male residents of the industrialized county seemed to be more susceptible to health effects of industrialisation, with significantly less increased life expectancy (0.123 years less per year) than male residents of the reference county. Female residents seemed to be more resistant to the health effects of industrialisation. No research study in the literature has reported such gender differences, and this finding warrants further investigation into the mechanisms resulting in these effects.

Research studies on air pollution, which is likely to result from industrialisation, have shown contradictory information on gender and air pollution. A recent review study of air pollution indicated that air pollution has stronger health effects among women than among men [26]. However, the mechanism resulting in the gender differences is still not clear. Most of these studies attributed the decreased physical functions, such as lung function, to gender differences in physiology, activities, coexposures, behaviors, and occupations. However, most of these studies examined only the incidence of respiratory symptoms among men and women, and did not point out whether these symptoms would lead to actual changes in life expectancy between the genders.

Research in gender inequality has generally agreed that women experience more ill health but have lower rates of mortality [26,27]. Gender inequality in health is commonly attributed to differing exposure, resources, and lifestyle, with men more likely than women to smoke and consume alcohol, and women more likely than men to be physically inactive [28-30]. However, whether men in Taiwan are more vulnerable or women in Taiwan have greater resistance to environmental changes due to industrialisation, the gender effect of industrialisation requires further investigation.

### Industrialisation and economic growth

Industrialisation itself, like all forms of economic growth, exerts intrinsically negative population health effects among those communities most directly involved in the transformations which it entails [1]. This is true for studies that have been published on populations in the western countries [31]. The current study’s findings provide evidence that in addition to suffering from a smaller increase in life expectancy, residents of the industrialized county did not experience any greater increase in individual income than did residents of the reference county. Taiwan, like many countries in the world, exchanges people’s health for economic growth through entering the industrialisation process [1]. However, while the literature discusses lessons learned from history regarding the effect of economic exchange and health risks resulting from industrialisation, the current study’s findings further raise the issue for the Taiwanese government regarding who shall benefit from the economic growth related to industrialisation.

### Limitations

The influence of industrialisation can be examined from many different perspectives, including health effects, economic effects, and effects on natural resources. The current study examined only one major health and economic outcome, and so may miss some other aspects of industrialisation. Furthermore, the impact of industrialisation may differ by industry type and outcomes. This study’s findings also may not be generalizable to the development of industries other than high-emission industries.

## Conclusions

The petrochemical industry is one of the fastest growing industries in East Asia, fuelled by the region’s rapid economic development. The growth of the petrochemical industry in Taiwan benefits not only the huge direct players but also the nation’s economic growth. Our study pointed out an environmental justice issue related to the development of such high-emission industry, which Taiwan’s government faces and which is shared by other countries that plan to or have already embraced petrochemical industrialisation.

## Competing interests

The authors declare that they have no competing interests.

## Authors’ contributions

YM has made substantial contributions to acquisition of data, and interpretation of data of this study and have been involved in drafting the manuscript and revising it critically for important intellectual content. WY has conducted analysis of data of this study and have been involved in drafting in manuscript and manuscript revision. CC has made substantial contributions to conception and design of the study have given final approval of the version to be published. All authors read and approved the final manuscript.

## Pre-publication history

The pre-publication history for this paper can be accessed here:

http://www.biomedcentral.com/1471-2458/14/247/prepub
